# A cross-sectional ecological analysis of international and sub-national health inequalities in commercial geospatial resource availability

**DOI:** 10.1186/s12942-018-0134-z

**Published:** 2018-05-23

**Authors:** Winfred Dotse-Gborgbortsi, Nicola Wardrop, Ademola Adewole, Mair L. H. Thomas, Jim Wright

**Affiliations:** 1 Kibi Government Hospital, Ghana Health Service, Accra, Ghana; 2 Geography and Environment, University of Southampton, Highfield, Southampton, SO17 1BJ UK

**Keywords:** Geocoding, Drive-times, Patient travel, Neighbourhood statistics, Digital divide, Geospatial data, Health inequalities, Inverse care law, GIS

## Abstract

**Background:**

Commercial geospatial data resources are frequently used to understand healthcare utilisation. Although there is widespread evidence of a digital divide for other digital resources and infra-structure, it is unclear how commercial geospatial data resources are distributed relative to health need.

**Methods:**

To examine the distribution of commercial geospatial data resources relative to health needs, we assembled coverage and quality metrics for commercial geocoding, neighbourhood characterisation, and travel time calculation resources for 183 countries. We developed a country-level, composite index of commercial geospatial data quality/availability and examined its distribution relative to age-standardised all-cause and cause specific (for three main causes of death) mortality using two inequality metrics, the slope index of inequality and relative concentration index. In two sub-national case studies, we also examined geocoding success rates versus area deprivation by district in Eastern Region, Ghana and Lagos State, Nigeria.

**Results:**

Internationally, commercial geospatial data resources were inversely related to all-cause mortality. This relationship was more pronounced when examining mortality due to communicable diseases. Commercial geospatial data resources for calculating patient travel times were more equitably distributed relative to health need than resources for characterising neighbourhoods or geocoding patient addresses. Countries such as South Africa have comparatively high commercial geospatial data availability despite high mortality, whilst countries such as South Korea have comparatively low data availability and low mortality. Sub-nationally, evidence was mixed as to whether geocoding success was lowest in more deprived districts.

**Conclusions:**

To our knowledge, this is the first global analysis of commercial geospatial data resources in relation to health outcomes. In countries such as South Africa where there is high mortality but also comparatively rich commercial geospatial data, these data resources are a potential resource for examining healthcare utilisation that requires further evaluation. In countries such as Sierra Leone where there is high mortality but minimal commercial geospatial data, alternative approaches such as open data use are needed in quantifying patient travel times, geocoding patient addresses, and characterising patients’ neighbourhoods.

**Electronic supplementary material:**

The online version of this article (10.1186/s12942-018-0134-z) contains supplementary material, which is available to authorized users.

## Background

Sustainable Development Goal (SDG) 3, Target 3.8 seeks to ‘achieve universal health coverage, including financial risk protection, access to quality essential health-care services…for all’ [1], with a similar target 3.7 seeking to deliver universal maternal healthcare coverage. GIS has been proposed as an integrative information and communication technology tool for accelerating progress towards universal health coverage (UHC) [2]. Informed decision-making is central to achieving UHC and spatial analysis enables precise identification of health needs to inform system strengthening interventions and can help to identify localised gaps in service provision, masked by national or provincial averages [2]. Supporting UHC has been proposed as a means of reducing mortality. For example, increasing the proportion of births attended by a skilled birth attendant at primary healthcare facilities can contribute to reduced maternal mortality [3].

To realise the potential of GIS, it has been argued that the health sector has to ‘geoenable’ its health information systems [2]. ‘Geoenabling’ entails putting in place the necessary governance structures, technical capacity, guidelines, standards, protocols, technology and core data to harness GIS’ potential. Thus, a management structure that provides sufficient funding to underpin GIS adoption, resources for creating and maintaining health information systems, and an underlying national spatial data infrastructure are all prerequisites for GIS uptake in the health sector. Awareness of GIS use in healthcare planning remains low even in developed countries [4], where within the UK National Health Service its use remains largely restricted to mapping.

Here, we focus on one such potential barrier to GIS uptake in the health sector, namely the availability of core data. In representing population demand for healthcare and examining patient interactions with healthcare facilities, the use of several key commercial geospatial resources has become widespread in many developed countries. These geospatial resources include reference data sets and tools for geocoding the residential addresses of patients presenting at healthcare facilities [5, 6], and transportation data that enable patient travel times to be computed from place of residence to facility [7, 8]. They also include area statistics and geodemographic data sets, which provide insights into neighbourhood characteristics that may be associated with healthcare demand and utilisation [9–11].

Although such data resources are generally available in high income countries, in many low and middle income countries (LMICs), such data may be patchy in coverage, imprecise, or lacking altogether. Furthermore, in developed countries, national spatial data infra-structures (SDIs) typically enable national mapping and statistical agencies to maintain address databases or dwelling frameworks, and thereby construct small area statistics. In LMICs, however, barriers relating to the global digital divide such as lack of financial resources, insufficient leadership and governance, poor internet bandwidth [12], lack of trained personnel, lack of vendor support, and power dynamics over information release all inhibit SDI development [13]. Even where open data initiatives exist as in the example of Kenya, such resources may still remain limited [14], although there is evidence [15, 16] that coverage of the world’s largest open geospatial database, OpenStreetMap (OSM), is rapidly expanding in many LMICs.

Over 30 years ago, an ‘inverse care law’ was first identified by Hart [17], which highlighted the frequently encountered perverse relationship between healthcare provision and need. Since then, there have been numerous studies that have quantified greater healthcare provision among areas of low need and lower healthcare provision in areas of high need [18–21], confirming this phenomenon in many settings. It is unclear whether data for planning healthcare delivery follow a similar pattern.

## Methods

### Aims

In this paper, we aim to quantify the extent to which the same perverse relationship with health needs applies to geospatial data availability as with healthcare provision. We explore two scales through a cross-sectional, ecological study design. We firstly examine the relationship between geospatial data availability and health need as measured by all-cause mortality and mortality due to three groups of causes, globally at national level. We then consider the relationship between health need and geospatial data availability in two sub-national case studies from Ghana and Nigeria.

### Data

At international level, we examine the availability, by country, of three sets of commercial data resources that are central to understanding population demand for healthcare and spatial patterns of healthcare utilisation. These are geocoding tools for locating patients’ residences; transportation network resources for computing patient travel from place of residence to health facility; and area statistics for characterising the neighbourhoods where patients live. We excluded other commercial geospatial data resources not directly related to healthcare-seeking behaviour, such as remotely sensed imagery. To identify such resources, we used the search strategy in Additional file 1: Table S1. We included only geospatial data resources that met the following criteria:

- Related to more than five countries, thereby having an international rather than national or regional remit
- Were not derived exclusively from open data and were provided as a commercial service
- Did not duplicate data resources already included in our analysis (for example where there were several APIs based on the same underlying data resource)
- Provided published statements of data availability or quality by country.

Where necessary, we contacted data providers to request permission to use data availability or quality statements in our analysis, only including those where such permission was granted. The geospatial resources that met all these criteria were included in our analysis are shown in Table 1 (Additional file 1: Tables S2–S4 documents data resources that were excluded and reasons for this).

**Table 1:** Commercial geospatial data resources for geocoding patient addresses, estimating travel times, and characterising patients’ neighbourhoods

Geospatial resource	Description	Web link
*Geocoding*		
ESRI geocoding resources	Underpinning resources for geocoding via API, desktop and online software	https://developers.arcgis.com/rest/geocode/api-reference/geocode-coverage.htm
Pitney Bowes	Geocoding API	https://developer2.pitneybowes.com/docs/location-intelligence/v1/en/index.html#GeoCode/Geocode/LI_GGM_Geo_Geocoding.html#GGM_Geo_Geocoding__Geocoding_CountrySpecific
TomTom	Resources for geocoding API	https://developer.tomtom.com/market-coverage-1
MapBox	Resources for geocoding API	https://www.mapbox.com/geocoding/#data
Loqate	Resources for geocoding service	https://loqate.com/countries-covered/
*Patient travel*		
ESRI/HERE	Underpinning resources for travel time estimation via API, desktop and online software	https://doc.arcgis.com/en/arcgis-online/reference/network-coverage.htm
Google traffic/speed limits	Resources accessible via Google Maps API	https://developers.google.com/maps/coverage
iGeoloise TravelTime Platform	Resources for API for computing travel times via public transport and driving	http://docs.traveltimeplatform.com/overview/supported-countries/
TomTom	API resources for routing and drive-times	https://developer.tomtom.com/market-coverage-1
MapBox Directions API	Resources for travel time API	https://www.mapbox.com/api-documentation/pages/traffic-countries.html
*Neighbourhood characterisation*		
Michael Bauer	Area statistics covering topics such as population, age-sex structure, consumer lifestyles, unemployment and purchasing power	http://www.english.mb-research.de/market-data-overseas.html
Mosaic Global geodemographic resources	Area statistics based on consumer classification system	http://www.experian.co.uk/assets/business-strategies/brochures/Mosaic_Global_factsheet%5b1%5d.pdf
Cameo International	Area statistics based on consumer classification system	http://www.callcredit.co.uk/media/1287258/cameo%20global%20map.jpg
Maptitude	Spatially disaggregated demographic data that are more than headcounts	https://www.caliper.com/maptdata.htm

Alongside these resources, we used all-cause mortality by country for the most recent period (2000–2015) reported by the World Health Organisation (WHO) [22], as a general health outcome measure and thereby metric of healthcare need. We also separately examined the major WHO categorization of mortality: non-communicable diseases; injuries; communicable diseases, maternal, perinatal, and nutritional conditions for 183 countries.

### Analysis

#### International analysis

National mortality data from WHO were age-standardised to account for differences in population structure between countries. As dependent territories are not reported separately in WHO mortality data, these were excluded from our analysis.

We then generated commercial geospatial resource indicators by country as follows:

- *Geocoding resources* Since the published level of geocoding availability and quality via the Google Application Programming Interface (API) scarcely varied by country, we used the geocoding precision levels published by Pitney Bowes, TomTom, MapBox, Loqate, and the Environmental Systems Research Institute (ESRI).
- *Resources for computing patient travel times* To characterise availability and quality of resources for computing travel times via the Google API, we generated a composite index by summing reported data availability for cycling directions, walking directions, driving directions, speed limits and availability of a traffic layer. Each of these was scored as two for ‘good quality and availability’, one for ‘approximate data quality and availability’ and zero otherwise. ESRI/HERE data quality and availability was characterised by six levels, based on availability of traffic and speed limit data, and completeness of street network coverage, whilst TomTom resources were characterised by availability of traffic flows, traffic incidents, and online routing. We separately recorded the availability of a traffic layer via MapBox and availability of routing for car travel only or car travel and public transport via iGeoloise TravelTime.
- *Area statistics for characterising patients’ neighbourhoods* To quantify availability of neighbourhood statistics by country, we computed three measures. Firstly, as a measure of spatial data disaggregation, we used the mean population per areal unit (lower mean populations indicate a higher level of spatial disaggregation) in Michael Bauer data sets. Where no data were available from this provider for a given country, we used the national 2015 population estimate from the WHO mortality database. Secondly, for the most detailed geography available in the Michael Bauer data, we counted the number of areal attributes available per country, setting this to zero where no data were available. Finally, for each country, we identified whether only one or both the geodemographic classifications (i.e. CAMEO Worldwide and Mosaic Global) were available, alongside availability of Maptitude demographic data.

To examine the availability of these geospatial resources relative to healthcare need, as measured by standardised all-cause mortality and cause-specific mortality, we computed relative concentration indices and slope indices of inequality [23] for each of these measures of geospatial data availability using a tool from Public Health England [24]. In this context, the slope index of inequality measured the change in mortality relative to ranked geospatial data availability/quality, whilst the relative concentration index measured the mortality gradient against relative geospatial data availability/quality.

We also created a composite index of commercial geospatial resource quality/availability (geospatial resource index) by combining these various indicators. For each of the three index domains (geocoding resources, patient travel, and neighbourhood characterisation), we ranked each country from highest to lowest based on each of the above indicators, then summed these ranks, dividing the total by the maximum possible summed rank to give an index for each domain between 0 and 1. To avoid the index being dominated by indicator availability at domain level, we then summed the three domain index values. We regressed logged standardised mortality against the geospatial resource index, identifying as outliers in terms of data availability those countries with studentised residuals greater than two. We also calculated the correlation of the geospatial resource index with the percentage of internet users and gross domestic product (GDP) per capita for 2016 in each country [25].

#### Sub-national case studies

To examine sub-national geospatial commercial resource availability and quality, two sub-national case studies were conducted, one in Eastern Region, Ghana and the other in Lagos State, Nigeria. Both focussed on success rates for geocoding facility locations (health facilities and schools respectively). In the absence of robust district-level mortality estimates, both studies examined geocoding success rates relative to area deprivation at administrative level 2 (districts in Ghana or local government areas in Nigeria). In this context, we consider area deprivation to reflect ‘an area’s potential for health risk from ecological concentration of poverty, unemployment, economic disinvestment, and social disorganisation’ [26].

In Eastern Region, 984 health facility place-names from 25 districts were obtained from the Ghana Health Service routine data repository (DHIMS2) and geocoded via an interface to the Google Maps API Version 2 [27]. Geocoding success was measured as the proportion of facilities per district for which a location within Eastern Region was returned. District deprivation was assessed firstly via the 2017 UNICEF District League Table (DLT) [28], a composite index of district development based on indicators of education, sanitation, rural water, health, security and governance. Secondly, district deprivation was also assessed via a bespoke district deprivation index. The bespoke deprivation index was created from 12 indicators representing six domains: information access, education, energy, employment, water and sanitation, and living conditions, adapting an approach used in South Africa [29]. Indicators values were drawn from 2010 census data [30]. Within each domain, each indicator was standardised by conversion to a z-score, with z-scores averaged for each domain. The average scores for the six domains were then summed to give a composite deprivation score.

Similarly, in Lagos State 310 schools, both private and public, from 20 Local Government Areas (LGAs) were obtained from online news media [31]. These were then geocoded using the Google Maps API Version 2 via BatchGeo [32]. A deprivation index with the same six domains as Ghana was created for the LGAs, but with 9 indicators drawn from 2006 census data acquired from the National Population Commission. These were then standardised and combined using the same method as for Eastern Region. For both case studies, geocoding success per district/LGA was then plotted against deprivation. Relative concentration indices and slope indices of inequality were computed for district-level geocoding success rates versus the deprivation measures.

## Results

### Characteristics of commercial geospatial data resources

Table 2 summarises the availability and precision of commercial geospatial resources for the 183 countries for which data were available in the WHO mortality database. There is considerable international variation in each indicator’s availability, with for example both predictive and live traffic data underpinning ESRI’s drive-time calculations in 11 countries, but conversely only partial coverage of the major road network being available in 24 countries. Similarly, market and demographic statistics were available for areas with average populations of less than a thousand in some countries, but over ten million in others.

**Table 2:** Geospatial resource availability and precision for 183 countries (portions of this table are modifications based on work created and shared by Google and used according to terms described in the Creative Commons 3.0 Attribution License. Used with permission. Copyright © 2017 Esri, ArcGIS Online, HERE, Increment P, GlobeTech and the GIS User Community. All rights reserved. © 2017 Michael Bauer Research GmbH, © TomTom 2018)

Availability and precision of geospatial data resources	No of countries (%)
*Geocoding*	
ESRI	
Level 1: address searches likely to result in either precise coordinates or interpolated location along street for address	42 (23.0%)
Level 2: address searches often result in precise coordinates or interpolated location along street, but sometimes street-level coordinates or coarser	16 (8.7%)
Level 3: address searches sometimes result in precise coordinates or interpolated location along street, but more often street-level coordinates or coarser	51 (27.9%)
Level 4: address searches result in imprecise locations, e.g. centroids of higher-level administrative boundaries	74 (40.4%)
Pitney Bowes Geocoding (highest precision available)	
Precise address point geocoding	20 (10.9%)
Address geocoding	39 (21.3%)
Street-level geocoding	60 (32.8%)
Post code	34 (18.6%)
Administrative boundaries or place-names	29 (15.8%)
Not specified	1 (0.5%)
TomTom geocoding (highest precision available)	
Address point	42 (23.0%)
Interpolated address	20 (10.9%)
Street-level	77 (42.1%)
Locality	44 (24.0%)
MapBox geocoding (highest precision available)	
Address geocoding	25 (13.7%)
Postcode	20 (10.9%)
Place-name	60 (32.8%)
No service	79 (42.6%)
Loqate geocoding	
Premises—point	52 (28.4%)
Premises	44 (24.0%)
Thoroughfare	68 (37.2%)
Locality	19 (10.4%)
*Patient travel*	
ESRI/HERE travel times	
Predictive traffic: comprehensive street data with live, historic, and predictive traffic	11 (6.0%)
Live traffic: comprehensive street data with live and historic traffic	40 (21.9%)
Historical traffic: comprehensive street data with historic traffic only	20 (10.9%)
Posted speed limits: comprehensive street data but with time-invariant travel times derived from speed limits	15 (8.2%)
Limited street coverage: partial street data for major roads only without minor or secondary roads; time-invariant travel times derived from speed limits	73 (39.9%)
Minimal street coverage: partial street data for some major roads only; no ground verification of network; no speed limit data	24 (13.1%)
Google travel times—traffic	
Traffic layer—available with good data quality and availability	91 (49.7%)
Traffic layer—available with approximate data quality or availability	1 (0.5%)
Traffic layer not available	91 (49.7%)
Google travel times—speed limits	
Speed limits—available with good data quality and availability	11 (6.0%)
Speed limits—available with approximate data quality or availability	166 (90.7%)
Speed limits not available	6 (3.3%)
Google travel times—cycling	
Cycling directions available	21 (11.5%)
Cycling directions unavailable	162 (88.5%)
iGeolise TravelTime Platform	
Travel times for public transport and driving	23 (12.6%)
Travel times for driving only	3 (1.6%)
Travel times unavailable	157 (85.8%)
TomTom	
Online routing with traffic incidents and traffic flows	42 (23.0%)
Online routing with traffic flows only	15 (8.2%)
Online routing without traffic	57 (31.1%)
No online routing	69 (37.7%)
MapBox	
Traffic layer available	33 (18.0%)
Traffic layer unavailable	150 (82.0%)
*Neighbourhood characterisation*	
Michael Bauer neighbourhood statistics	
Number of areal attribute groups per country—global mean (5th centile; 95th centile)	4 (0; 9)
Mean population per areal unit by country—global median (5th centile; 95th centile)	130,000 (411; 23,801,400)
*Mosaic Global*	
Geodemographic classification available	24 (13.1%)
Geodemographic classification unavailable	159 (86.9%)
*Cameo worldwide*	
Geodemographic classification available	39 (21.3%)
Geodemographic classification unavailable	144 (78.7%)
*Maptitude*	
Demographic data (beyond population headcounts) available	13 (7.1%)
Demographic data (beyond population headcounts) unavailable	170 (92.9%)

### International inequality in access to commercial geospatial data resources

Table 3 shows two health inequality metrics, the relative concentration index and slope index of inequality, for national all-cause mortality versus international availability and quality of various commercial geospatial data resources. Slope index of inequality values indicate the effect on all-cause mortality of moving from the most data-poor country to the most data-rich. Negative concentration indices suggest mortality is concentrated among data-poor populations, whilst zero indicates no mortality gradient relative to geospatial data. Slope indices of inequality were significantly different from zero for most sources of commercial geospatial data considered, suggesting significant health inequalities for most resources. However, levels of inequality were lower for resources for computing patient travel times than for resources for geocoding patient addresses or characterising patients’ areas of residence. For example, concentration indices for ESRI’s geocoding service and population size of Michael Bauer’s areal units were − 0.14 and − 0.12 respectively, whereas concentration indices for Google and ESRI’s patient travel resources were less than − 0.07.

**Table 3:** Metrics of inequality in international availability of commercial geospatial data resources, relative to age-standardised all-cause mortality for 2015 in 183 countries (portions of this table are modifications based on work created and shared by Google and used according to terms described in the Creative Commons 3.0 Attribution License. Used with permission. Copyright © 2017 Esri, ArcGIS Online, HERE, Increment P, GlobeTech and the GIS User Community. All rights reserved. © 2017 Michael Bauer Research GmbH, © TomTom 2018)

Index domain	Commercial geospatial resource availability/quality measure	Relative concentration index	Slope index of inequality (95% confidence intervals)
Geocoding	ESRI	− 0.14	7.54 (6.47–8.61)
	Pitney Bowes	− 0.04	2.18 (0.66–3.69)
	TomTom	− 0.02	0.9 (− 0.70 to 2.49)
	MapBox	− 0.07	4.24 (2.77–5.71)
	Loqate	− 0.01	0.45 (− 1.12 to 2.03)
	Geocoding domain	− 0.07	3.21 (1.81–4.62)
Patient travel	ESRI / HERE	− 0.03	1.55 (0.05–3.06)
	Google Maps	− 0.06	3.24 (1.75–4.72)
	MapBox	− 0.07	7.79 (5.88–9.70)
	iGeolise TravelTime	− 0.05	7.25 (4.89–9.61)
	TomTom	− 0.01	0.32 (− 1.27 to 1.90)
	Patient travel domain	− 0.04	1.93 (0.47–3.39)
Neighbourhood characterisation	Michael Bauer—average population per areal unit	− 0.12	6.08 (4.89–7.27)
	Michael Bauer—no. of areal attribute groups	− 0.07	3.54 (2.14–4.94)
	Geodemographic classification/demographic data availability (Experian Global & Cameo Worldwide; Maptitude)	− 0.10	5.91 (4.56–7.25)
	Neighbourhood characterisation domain	− 0.13	6.39 (5.24–7.54)
Overall index	Overall commercial geospatial resource quality/availability index	− 0.08	4.01 (2.64–5.37)

Table 4 shows the inequality metrics broken down by the WHO cause-specific mortality categorization. As indicated by the concentration index values differing from zero, measured inequalities were greatest for the communicable disease group and lowest for non-communicable diseases, both for the overall index and for the geocoding and neighbourhood characterisation domains. Concentration index values and therefore measured inequality were closer to zero for the patient travel domain, as with all-cause mortality.

**Table 4:** Metrics of inequality in international availability of commercial geospatial data resources, relative to age-standardised cause-specific mortality for 2015 in 183 countries

Commercial geospatial resource availability/quality measure	Relative concentration index	Slope index of inequality (95% confidence intervals)
*Relative to mortality from communicable, maternal, perinatal and nutritional conditions*		
Geocoding domain	− 0.121	3.05 (0.82–5.27)
Patient travel domain	− 0.020	0.52 (− 1.77 to 2.80)
Neighbourhood characterisation domain	− 0.335	8.44 (6.53–10.35)
Overall geospatial resource index	− 0.159	4.01 (1.81–6.20)
*Relative to mortality from non*-*communicable diseases*		
Geocoding domain	− 0.040	1.38 (0.84–1.91)
Patient travel domain	− 0.034	1.18 (0.63–1.73)
Neighbourhood characterisation domain	− 0.056	1.91 (1.41–2.41)
Overall geospatial index	− 0.045	1.54 (1.01–2.06)
*Relative to mortality from injuries*		
Geocoding domain	− 0.092	0.39 (0.22–0.56)
Patient travel domain	− 0.034	0.14 (− 0.03 to 0.32)
Neighbourhood characterisation domain	− 0.170	0.72 (0.58–0.87)
Overall geospatial index	− 0.103	0.44 (0.27–0.60)

### International geospatial resource index

**Fig. 1:**
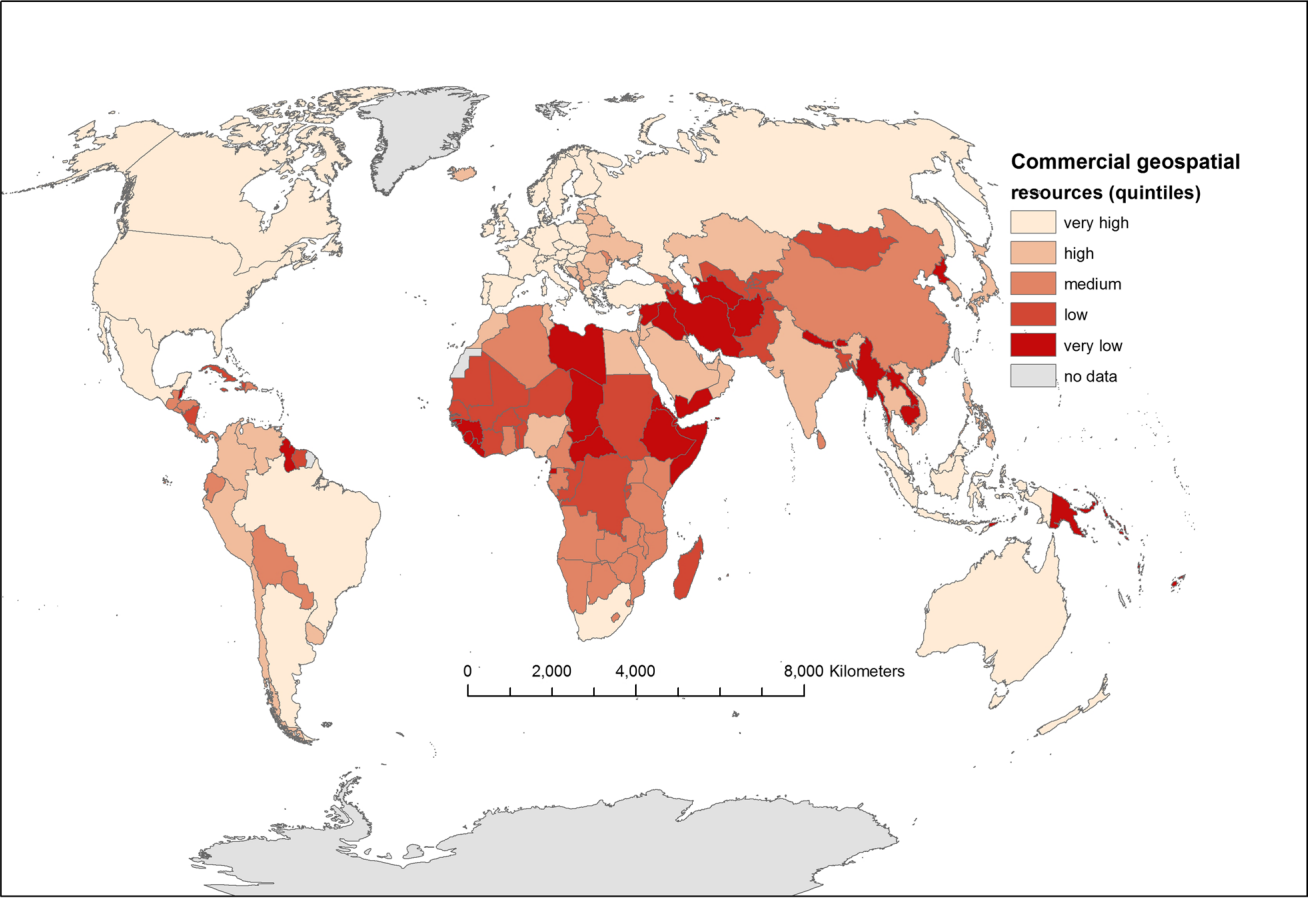
An index of commercial geospatial resource quality/availability for healthcare planning by country (based on a Winkel-Tripel projection. Portions of this graphic are modifications based on work created and shared by Google and thematicmapping.org and used according to terms described in the Creative Commons 3.0 Attribution License. Used with permission. Copyright © 2017 Esri, ArcGIS Online, HERE, Increment P, GlobeTech and the GIS User Community. All rights reserved. © 2017 Michael Bauer Research GmbH, © TomTom 2018)

Figure 1 shows the international geospatial resource index (illustrating quality/availability of commercial geospatial data resources for healthcare planning). According to the composite index, the quality/availability is generally high in the Americas, Australasia and Europe, but low in Africa and south Asia. However, some data providers document potentially valuable geospatial resources for healthcare planning in countries with high mortality, particularly in west and southern Africa. For example, a traffic layer is available via Google and interpolated street address level geocoding is documented by Pitney Bowes for Nigeria, both potentially valuable in understanding patient travel.

The geospatial resource index was strongly correlated with the percentage of internet users per country in 2016 (r = 0.77, p < 0.001, n = 183) and to a lesser extent with GDP per capita (r = 0.68, p < 0.001, n = 174). Several African countries such as South Africa and Mozambique had comparatively high geospatial data availability/quality scores given GDP, whilst several of the Gulf States (e.g. Qatar, United Arab Emirates) and smaller island states (e.g. Iceland, the Seychelles) had comparatively low index values given their GDP per capita. Similar patterns were observable for index values versus internet use.

**Fig. 2:**
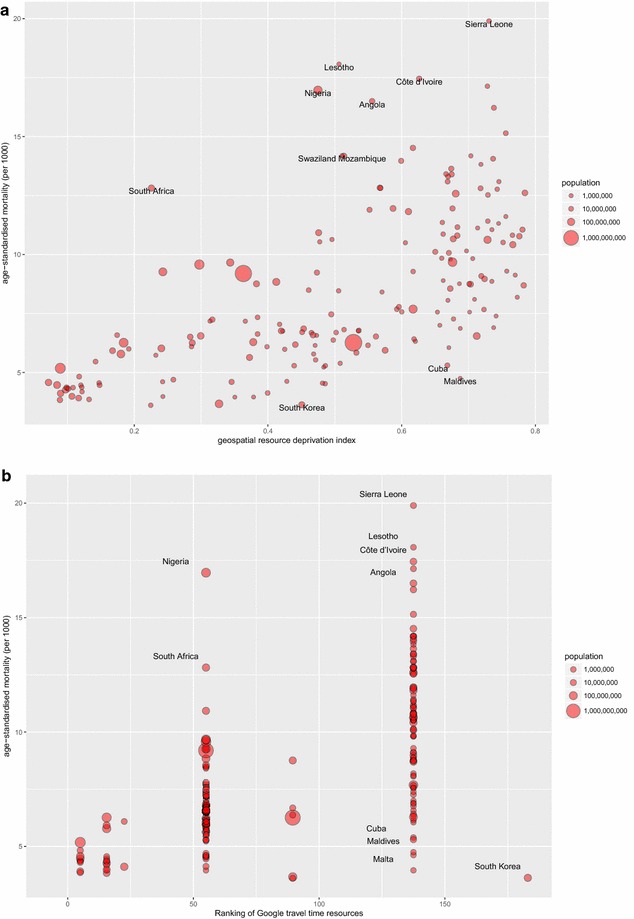
Age-standardised all-cause mortality for 2015 versus **a** an index of commercial geospatial resource quality/availability; **b** ranked availability of Google Maps travel time resources (labelled countries were identified as outliers. Portions of this graphic are modifications based on work created and shared by Google and used according to terms described in the Creative Commons 3.0 Attribution License. Used with permission. Copyright © 2017 Esri, ArcGIS Online, HERE, Increment P, GlobeTech and the GIS User Community. All rights reserved. © 2017 Michael Bauer Research GmbH; © TomTom 2018)

Figure 2 shows the distribution of standardised all-cause mortality in relation to the geospatial resource index. As anticipated from the deprivation indicators above, the pattern of all-cause mortality broadly follows geospatial resource quality/availability. Several outliers are labelled in Fig. 2. Countries with low all-cause mortality and low commercial geospatial data resources were typically either small island states such as Malta and the Maldives, or states with strict controls on international transfers of national data, such as South Korea and Cuba. South Africa was notable for its high all-cause mortality but comparatively high commercial geospatial resource availability, with similar outliers being in southern or west Africa.

### Sub-national case studies

**Fig. 3:**
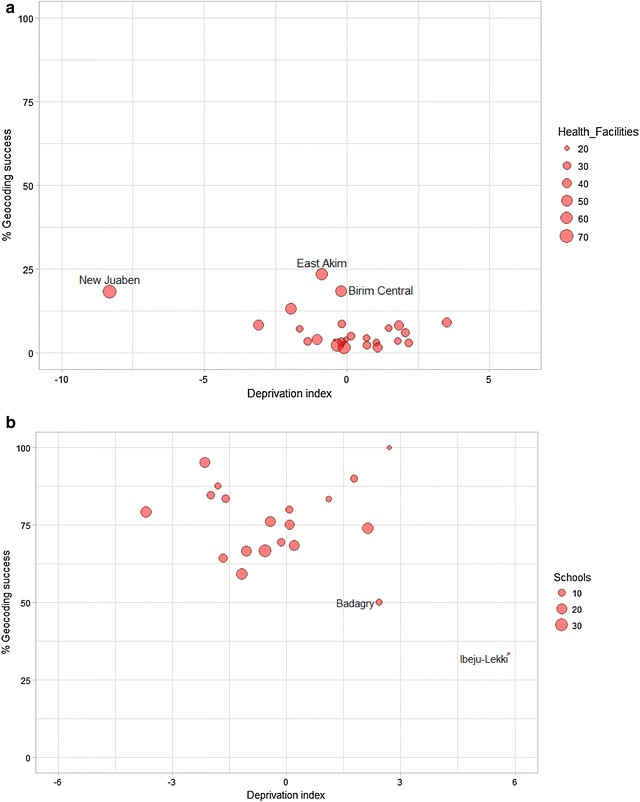
Deprivation score and geocoding success rates for **a** health facilities in 25 districts of Eastern Region, Ghana and **b** schools in 20 local government areas in Lagos State, Nigeria

Figure 3 shows the relationship between geocoding success rate and deprivation, in Lagos State and Eastern Region, with no clear relationship emerging overall. Geocoding success rates were low for health facilities in Eastern Region, but much higher for schools in Lagos State. In Eastern Region, districts such as East Akim and Birim Central had high success rates although their deprivation score was close to the average. Further exploration revealed these two districts had the highest number of hospitals (4 each) in the region and hospitals had the highest geocoding success rate (70.6%) compared with other health facility types. Likewise, the regional capital of New Juaben with 3 hospitals, was least deprived and had a high geocoding success rate. In Nigeria, two LGAs containing a small number of schools, Badagry and Ibeju-Lekki, were more deprived and had lower geocoding success rates, particularly influencing the observed relationship between deprivation and geocoding success.

Table 5 shows inequality metrics for geocoding success rates versus area deprivation at the LGA or district level in Lagos State, Nigeria, and Eastern Region, Ghana. There was no evidence of inequality in geocoding success relative to area deprivation in Lagos State, as indicated by the concentration index of zero. In Ghana, evidence for lower geocoding success in more deprived areas was mixed. When the DLT was used to measure area deprivation, confidence intervals for the slope index of inequality straddled zero, indicating no significant inequality. When the bespoke area deprivation index was used, the slope index of inequality was significantly different from zero.

**Table 5:** Inequalities in geocoding success rates, relative to area deprivation (for 984 health facilities in 25 districts in Eastern Region, Ghana and 298 schools in 20 LGAs in Lagos State, Nigeria)

Case study details	Relative concentration index	Slope index of inequality (95% confidence intervals)
*Eastern Region, Ghana*		
Geocoding success rate for health facilities		
Relative to UNICEF District League Table	0.14	6.87 (− 2.24 to 15.97)
Relative to bespoke district deprivation index	0.20	9.57 (0.93–18.21)
*Lagos State, Nigeria*		
Geocoding success rate for schools		
Relative to LGA deprivation index	0.00	− 1.99 (− 24.41 to 20.43)

## Discussion

To our knowledge, our analysis is the first to examine global patterns of commercial geospatial data availability in relation to health outcomes. As observed with healthcare services, both internationally and for two sub-national case studies, these data are inversely correlated with health need, as measured by mortality and deprivation respectively. This disparity in geospatial data availability is more pronounced for mortality due to communicable diseases. Such data are thus frequently unavailable for planning healthcare provision or geocoding cases for widespread communicable diseases such as malaria. The availability of commercial geospatial data resources broadly follows the same pattern as that identified in analyses of the global digital divide, with for example sub-Saharan Africa being the world region lagging furthest behind North America in internet users [33] and Africa having the lowest Information and Communication Technology Development Index [34]. The pattern of outliers is somewhat different to these more general analyses of the global digital divide, however. For example, an assessment of digitalisation relative to GDP per capita [35] indicated lower than anticipated digitalisation in Oman and Kuwait but higher digitalisation in South Korea. We observed the same relationship for the Gulf States in relation to our commercial geospatial index but low international availability of commercial geospatial data in South Korea. However, because of restrictions on the export of mappable data out of the country, South Korea has previously been reported as lacking data from major providers such as Google [36].

In the absence of such commercial tools, and where sufficient capacity exists, researchers in LMICs have resorted to alternative strategies for geocoding data, computing drive-times, and characterising patients’ places of residence. Where the human resources, infrastructure, and tools exist, one geocoding strategy is to rely on open data, particularly OSM, as has been attempted in Thailand and Mozambique for healthcare management [37, 38]. Elsewhere, a study in Yemen, relied on direct measurement of drive-times taken on specific routes [39]. A Kenyan study explored participatory mapping and use of local landmarks as strategies for geocoding patient addresses [40], whilst in a Mexican study, a software application was developed that allowed patients to identify their place of residence through interpretation of Google Earth and StreetView imagery [41]. In Cote d’Ivoire, aggregated call record data from mobile phones have been used to develop a proxy for regional socio-economic indicators [42], whilst in Accra, vegetation metrics derived from QuickBird satellite imagery were correlated with a slum index [43]. Without such innovative geocoding or neighbourhood characterisation strategies, there is potential for misclassification of neighbourhood characteristics [44] and environmental exposures [45] when analysing LMIC patient data using inexact geocodes or areal statistics relating to large populations.

International organisations have invested heavily in geospatial infra-structure, capacity-building, and technology to address this paucity of commercial data and its affordability in low-resource settings. The WHO for example has developed AccessMod to estimate patient travel times to health facilities via a cost surface approach [46]. Because of limited access to software and technical GIS skills, WHO also developed the HealthMapper software, which packages public domain spatial databases with a user-friendly interface to broaden uptake of GIS for healthcare planning. HealthMapper has been used in schistosomiasis control [47] and prioritising areas for filariasis elimination [48]. More recently, Measure Evaluation have supported health management information systems by developing the Place Mapping plug-in for QGIS to ease handling and display of point data sets [49] and provided guidance on diagnosing positional errors [50].

We found less conclusive evidence that geocoding success rates were lower in deprived areas when considering the two sub-national case studies of Lagos State, Nigeria and Eastern Region, Ghana. There was no evidence of inequality in geocoding success in Lagos State, but mixed evidence of such inequality in Eastern Region. However, elsewhere an apparent inverse relationship between geocoding outcomes and area deprivation has been observed at local level in other LMICs. A study in the Brazilian city of Belo Horizonte, for example, found that geocoding precision via the Google geocoding API was lower in slum areas than in formal urban neighbourhoods [51]. In response to this issue, the What3Words georeferencing system, which uses an algorithm to assign three words as a unique, human-friendly georeference to each of 57 trillion grid squares globally, has been used to locate addresses in Brazilian favelas lacking conventional addressing systems [52].

Our findings are subject to several limitations. Our study assumes that the data provider’s published country coverage information is an accurate reflection of geospatial data availability and precision across all countries. In reality, export of geospatial data from one country to another may be restricted by trade embargos, as has previously happened with satellite imagery exports to India for example [53], and where commercial geospatial data are available internationally, they may be unaffordable within the national health sector. In assessing the international availability of geodemographic classifications, we focussed on two major international data providers only, potentially omitting smaller data providers operating in individual countries. However, a recent study of the international availability of geodemographic classifications [54] showed very similar patterns to that found here. Computed inequality indices are also likely to be lower for metrics of geospatial data availability and precision based on a small number of ordinal classes (e.g. for geodemographic data availability), than metrics on a ratio scale (e.g. mean population per areal unit). In our sub-national case studies, geocoding success rates for higher tier facilities (e.g. hospitals or large secondary schools) may be higher than for lower tier facilities (e.g. primary care facilities such as Community-Based Health Planning and Services compounds). Since such facilities are more often found in urban areas, such heterogeneity in facility type may lead to an over-estimate of inequality in geocoding resource access.

Given the rapid pace of change in the geospatial data sector, this analysis could be repeated in the future to monitor rapidly changing data availability in relation to health outcomes. We have only considered the relationship between commercial geospatial resources and mortality, but geospatial resource availability could also be examined in relation to underlying drivers of health outcomes, such as country income levels, internet access, and relevant government policies, or in relation to measures relevant for other sectors (such as infrastructure). There would also be scope to combine the country-level results presented here on commercial geospatial data with recent assessments of OSM completeness by country [15, 16]. These studies, based on stratified assessment of OSM road completeness [16] or saturation of user contributions [15], suggest that global commercial geospatial resources and OSM completeness patterns are somewhat different, with for example, China and Egypt having low OSM completeness [16]. The potential utility of commercial geospatial data resources for healthcare planning could also be explored through a case study country (e.g. South Africa), deliberately selected because of its high mortality and high geospatial data availability.

The comparatively richer commercial resources in west and southern African countries such as Nigeria and South Africa merit further investigation for healthcare planning in these countries, subject to sufficient funds being available to support their use in a given project.

In contrast to analyses from elsewhere [51], our sub-national analyses in Lagos State suggested only limited evidence that geocoding success was lower in deprived areas. Our inconclusive results may be because the large areal units we used to assess deprivation may mask localised pockets of deprivation, which have been identified via previous work in Accra [55]. In our analysis, we implicitly use geocoding success rates to assess spatial variation in the Google Maps API reference data set and geocoding algorithm performance. However, geocoding success also depends on the quality of the input address data [56] and this will have been affected by local variations in service provision tiers, language of place-names and place-name aliases in our Nigerian and Ghanaian case studies. Furthermore, in our two case studies, success rates in geocoding service locations in Eastern Region, Ghana, and Lagos State, Nigeria were very different, despite identical documented levels of geocoding service precision. Despite their growing potential, this suggests careful evaluation of such resources is needed prior to their application to healthcare management.

In many other countries with high mortality, commercial geospatial data availability was very low. By examining commercial geospatial resource availability alongside OSM completeness [16], such countries could be targeted via non-profit initiatives to increase the availability of open geospatial data availability, such as those undertaken through OSM-based volunteer mapping initiatives by humanitarian organisations [57, 58]. Whilst commercial geospatial resource availability may improve in these countries in the future, in the interim, there remains a need for innovative solutions to geocoding outpatient data, estimating patient travel times and characterising neighbourhoods in such countries as described above.

## Conclusions

To our knowledge, our analysis is the first to examine global patterns of commercial geospatial data availability in relation to health outcomes. The relationship observed between commercial geospatial resource availability and health needs suggests LMICs still have inadequate geospatial resources for the type of granular analysis needed to drive the SDG agenda surrounding UHC. This inequality in data availability is more pronounced for mortality due to communicable diseases than for all-cause mortality. There were some outliers, however: several west and southern African countries such as Nigeria and South Africa had comparatively high geospatial data availability and high mortality. In contrast, there were several countries with low mortality and comparatively geospatial data availability, often island states (e.g. the Maldives) or those with policy restrictions on geospatial data (e.g. South Korea). This analysis thus suggests some resources, particularly those for quantifying patient travel times, are penetrating countries with high all-cause mortality. In countries such as South Africa and Nigeria where there is high mortality but also comparatively rich commercial geospatial data, these data are a potential resource for examining healthcare utilisation that requires further evaluation. In countries such as Sierra Leone where there is high mortality but minimal commercial geospatial data, alternative approaches, for example based on open data such as OSM, are needed in quantifying patient travel times, geocoding patient addresses, and characterising patients’ neighbourhoods.

In many instances, our examination of patterns of commercial geospatial availability confirms other studies of global digitalisation, with for example lower levels of digitalisation in the Gulf States for a given level of GDP per capita. However, even where this is comparatively high availability of relevant commercial geospatial data availability, this alleviates just one barrier among many that inhibit uptake of GIS in healthcare planning. Beyond increasing the availability of core data, further investments are needed in technical capacity-building, awareness-raising, guidelines, standards and protocols if the potential of such data is to be realised within the health sector.

## Additional file

**Additional file 1.** Search strategy for identifying relevant commercial geospatial data resources and details of data resources excluded from the analysis.

## Abbreviations

API: Application Programming Interface
DLT: District League Table
DHIMS: District Health Information Systems
ESRI: Environmental Systems Research Institute
GDP: gross domestic product
LGA: local government area
LMICs: low and middle-income countries
OSM: OpenStreetMap
SDGs: Sustainable Development Goals
SDI: spatial data infra-structure
UHC: universal health coverage
WHO: World Health Organisation
