# Supplementary material for: A cross-sectional ecological analysis of international and sub-national health inequalities in commercial geospatial resource availability
Source: Int J Health Geogr. 2018 May 23;17:14. doi: 10.1186/s12942-018-0134-z (PMC5966850; doi:10.1186/s12942-018-0134-z)
Supplement: Supplementary file 1 — Additional file 1. Search strategy for identifying relevant commercial geospatial data resources and details of data resources excluded from the analysis. [file 12942_2018_134_MOESM1_ESM.docx]

| **Commercial Geospatial Resource Domain** | **Search terms** |
| --- | --- |
| Geocoding | *‘world geocoding’ OR ‘global geocoding’ OR ‘geocoding API’ OR ‘international geocoding’* |
| Patient travel | *‘routing API’ OR ‘directions API’ OR ‘drive-time API’ OR ‘travel time API’* |
| Neighbourhood characterisation | *‘commercial demographic data’ OR ‘commercial demographic data international coverage’ OR ‘global geodemographic data’ OR ‘geodemographic segmentation data’* |

Table S1: Search terms used to identify commercial geospatial resources from three domains with application to healthcare management (searches conducted via Google during December 2017 and January 2018.

| **Data Source** | **Web link** | **Status** | **Reasoning for inclusion/exclusion** |
| --- | --- | --- | --- |
| OpenCageData | https://geocoder.opencagedata.com/api | Excluded | A commercial service that is based on open data. |
| Mapbox | https://www.mapbox.com/geocoding/#coverage  https://122e4e-mapbox.global.ssl.fastly.net/api-documentation/#introduction | Included | International coverage, commercial source, data quality and availability statements available. |
| ESRI/Here | https://developer.here.com/documentation/geocoder/topics/coverage-geocoder.html | Included | International coverage, commercial source, data quality and availability statements available. |
| JawgMaps | https://www.jawg.io/docs/apidocs/places/reverse/ | Excluded | A commercial service that is based on open data. |
| Sygic | <http://www.sygic.com/developers/maps-api-services/geolocation-and-search-api/geocode> | Excluded | Underlying reference data sources are not documented; country coverage also not documented. |
| Neutrino API | https://www.neutrinoapi.com/api/geocode-address/?gclid=EAIaIQobChMI7L_X5OiB2AIVS5PtCh25EgN8EAAYASAAEgK4s_D_BwE | Excluded | Underlying reference data sources are not documented; country coverage also not documented. |
| Graphopper | https://graphhopper.com/api/1/docs/geocoding/ | Excluded | A commercial service that is based on open data. |
| Pitney Bowes | https://locate.pitneybowes.com | Included | International coverage, commercial source, data quality and availability statements available. |
| Loqate | https://loqate.com/enhance/geocode/ | Included | International coverage, commercial source, data quality and availability statements available. |
| Geocodio | https://geocod.io | Excluded | Data covers less than six countries: USA and Canada only. |
| SmartyStreets | https://smartystreets.com/products/apis/international-street-api | Excluded | Underlying reference data sources are not documented; country coverage also not documented. |
| Carto | https://carto.com/docs/carto-engine/dataservices-api/geocoding-functions/ | Excluded | A commercial service that is based on open data. |
| Cartotype | http://www.cartotype.com/developers/documentation/35-geocoding-and-addresses | Excluded | Underlying data is based on open data. |
| PCA Predict | https://www.pcapredict.com/geocoding-service/api/ | Excluded | Duplicates data from other source that is already included in analysis**.** |
| Geocode Farm | https://geocode.farm | Excluded | International coverage, commercial source, data quality and availability statements available. However, partner to TomTom, so duplicated elsewhere. |
| Geoconcept | http://en.geoconcept.com/geocoding-api | Excluded | Duplicates data from other source that is already included in analysis**.** |
| Mappify | <https://mappify.io> | Excluded | Data covers less than six countries: Australia only. |
| TomTom Mapping | https://developer.tomtom.com/online-search/online-search-documentation-geocoding/geocode | Included | International coverage, commercial source, data quality and availability statements available |
| PickPoint | https://pickpoint.io/api-reference | Excluded | Underlying data is based on open data. |
| Melissa Global Intelligence | https://www.melissa.com/ca/global-location/geocoder.html | Included but no permission | International coverage, commercial source, data quality and availability statements available. |
| Unwired labs | https://unwiredlabs.com/geocoding-api | Excluded | Underlying reference data sources are not documented; country coverage also not documented. |

Table S2: Geocoding resources identified via searches and reasons for their inclusion or exclusion in the analysis

| **Data Source** | **Web link** | **Status** | **Reasoning for inclusion/exclusion** |
| --- | --- | --- | --- |
| iGeoloise Travel Time Platform | http://docs.traveltimeplatform.com/overview/introduction/ | Included | International coverage, commercial source, data quality and availability statements available. |
| ArcGIS / ESRI | https://developers.arcgis.com/javascript/3/jssamples/analysis_connector  iginstodestinations.html | Included | International coverage, commercial source, data quality and availability statements available. |
| WalkScore | https://www.walkscore.com/professional/travel-time-api.php | Excluded | Data covers less than six countries: USA, Canada, Australia and New Zealand only. |
| Mercator Geosystems | http://www.mercatorgeosystems.com/products/websites/ | Excluded | Data covers less than six countries: UK only. |
| Google Maps API | <https://developers.google.com/maps/coverage> | Included | International coverage, commercial source, data quality and availability statements available. |
| MapBox | https://www.mapbox.com/api-documentation/ | Included | International coverage, commercial source, data quality and availability statements available. |
| HERE Maps | <https://developer.here.com/documentation/routing/topics/resource-type-route-summary.html> | Excluded | International coverage, commercial source, data quality and availability statements available, but partner to ESRI, so duplicated elsewhere. |
| TomTom | https://developer.tomtom.com/market-coverage-1 | Included | International coverage, commercial source, data quality and availability statements available. |
| Open Source Routing Machine | http://project-osrm.org/docs/v5.10.0/api/#general-options | Excluded | A commercial service that is based on open data. |
| Geopointe | http://help.geopointe.com/customer/en/portal/articles/2371438-distance-service-api | Excluded | International coverage, commercial source, but data quality / availability statements refer to Google, so likely duplicate. |
| Map My India | http://www.mapmyindia.com/api/advanced-maps/doc/distance-api | Excluded | Data covers less than six countries: India only. |
| MapZen | https://mapzen.com/documentation/mobility/matrix/api-reference/ | Excluded | A commercial service that is based on open data. |

Table S3: Patient travel resources identified via searches and reasons for their inclusion or exclusion in the analysis

| **Data Source** | **Web link** | **Status** | **Reasoning for inclusion/exclusion** |
| --- | --- | --- | --- |
| Cameo International | http://www.callcredit.co.uk/products-and-services/consumer-marketing-data/segmentation-analysis/cameo-global-classifications] | Included | International coverage, commercial source, data quality and availability statements available. |
| Maptitude | https://www.caliper.com/maptdata.htm | Included | Commercial source, national mapping agencies and data from HERE |
| Living Atlas | https://livingatlas.arcgis.com/en/#s=0 | Excluded | Open source data. |
| geoTribe | http://rdaresearch.com/explorer | Excluded | Data covers less than 6 countries: Australia, New Zealand, the UK, the USA and Canada. |
| AllMapData | https://www.allmapdata.com/products/digital-map-data/geodemographics/mbr-socio-demographics/ | Excluded | Reseller of Michael Bauer data. |
| Michael Bauer | http://www.english.mb-research.de/market-data-overseas.html | Included | International coverage, commercial source, data quality and availability statements available. |
| GlobalDemographics | http://www.globaldemographics.com/databases/countries | Excluded | Data does not cover small areas. |
| Mosaic Global | http://www.experian.co.uk/assets/business-strategies/brochures/Mosaic_Global_factsheet%5b1%5d.pdf | Included | International coverage, commercial source, data quality and availability statements available. |
| Acorn through CACI | https://www.caci.co.uk/products/product/acorn | Excluded | Data covers less than 6 countries: the UK only. |
| Open Geodemographics | http://www.opengeodemographics.com | Excluded | Open source data and only covers the UK. |
| Patchwork Nation | http://www.patchworknation.org | Excluded | Open source data and only covers USA. |
| NUMAP | http://www.numaps.com.au/numaps/index.cfm/support/web-services-api/ | Excluded | Data covers less than 6 countries: Australia only. |
| AFD Software | http://www.afd.co.uk/data-sets/censation/ | Excluded | Data covers less than 6 countries: the UK only. |
| STI: Landscapes | http://www.synergos-tech.com/ls_intro.htm | Excluded | Data covers less than 6 countries: the USA only. |
| People and Places | http://www.p2peopleandplaces.co.uk | Excluded | Data covers less than 6 countries: the UK only. |
| Claritas | https://segmentationsolutions.nielsen.com/mybestsegments/Default.jsp?I  D=30&menuOption=segmentdetails&pageName=Segment%DEtails | Excluded | Data covers less than 6 countries: the USA only. |
| Acxiom Personicx | http://www.personicx.co.uk | Excluded | Data covers less than 6 countries: the UK only. |

Table S4: Neighbourhood charaterisation resources identified via searches and reasons for their inclusion or exclusion in the analysis
